# The Convergence Model of Brain Reward Circuitry: Implications for Relief of Treatment-Resistant Depression by Deep-Brain Stimulation of the Medial Forebrain Bundle

**DOI:** 10.3389/fnbeh.2022.851067

**Published:** 2022-04-01

**Authors:** Vasilios Pallikaras, Peter Shizgal

**Affiliations:** Center for Studies in Behavioral Neurobiology, Department of Psychology, Concordia University, Montreal, QC, Canada

**Keywords:** intracranial self-stimulation, dopamine, psychomotor stimulants, affective neuroscience, psychophysical inference

## Abstract

Deep-brain stimulation of the medial forebrain bundle (MFB) can provide effective, enduring relief of treatment-resistant depression. Panksepp provided an explanatory framework: the MFB constitutes the core of the neural circuitry subserving the anticipation and pursuit of rewards: the “SEEKING” system. On that view, the SEEKING system is hypoactive in depressed individuals; background electrical stimulation of the MFB alleviates symptoms by normalizing activity. Panksepp attributed intracranial self-stimulation to excitation of the SEEKING system in which the ascending projections of midbrain dopamine neurons are an essential component. In parallel with Panksepp’s qualitative work, intracranial self-stimulation has long been studied quantitatively by psychophysical means. That work argues that the predominant directly stimulated substrate for MFB self-stimulation are myelinated, non-dopaminergic fibers, more readily excited by brief electrical current pulses than the thin, unmyelinated axons of the midbrain dopamine neurons. The series-circuit hypothesis reconciles this view with the evidence implicating dopamine in MFB self-stimulation as follows: direct activation of myelinated MFB fibers is rewarding due to their trans-synaptic activation of midbrain dopamine neurons. A recent study in which rats worked for optogenetic stimulation of midbrain dopamine neurons challenges the series-circuit hypothesis and provides a new model of intracranial self-stimulation in which the myelinated non-dopaminergic neurons and the midbrain dopamine projections access the behavioral final common path for reward seeking via separate, converging routes. We explore the potential implications of this convergence model for the interpretation of the antidepressant effect of MFB stimulation. We also discuss the consistent finding that psychomotor stimulants, which boost dopaminergic neurotransmission, fail to provide a monotherapy for depression. We propose that non-dopaminergic MFB components may contribute to the therapeutic effect in parallel to, in synergy with, or even instead of, a dopaminergic component.

## Introduction

Major depressive disorder is among the most common mental illnesses, affecting 1 in 20 adults worldwide, and a leading cause of disability and suicide (Mathers, 2016; Friedrich, 2017). Depressive symptomatology is episodic and recurrent with lifetime relapse rates of 50%; some 80% of individuals who have had two major depressive episodes will experience at least a third (Burcusa and Iacono, 2007). Although multiple antidepressant interventions exist, about 3 in 10 individuals with major depressive disorder suffer from chronic symptoms that are unimproved after several rounds of conventional treatment (Gaynes et al., 2020). This condition, which entails serious societal and personal ramifications, is called treatment-resistant depression. In comparison to non-resistant depression, treatment-resistant depression is linked to higher hospitalizations and healthcare costs and to lower quality of life (Bergfeld et al., 2018; Gaynes et al., 2020). Importantly, the suicide attempt rate for treatment-resistant depression is at least double the lifetime rate in non-resistant depression and 15 times the rate in the normal population (Bernal et al., 2007).

In an effort to alleviate treatment-resistant depression, experimental interventions are often tried. A class of such interventions focuses on neuromodulation. Among them, deep brain stimulation is a neurosurgical approach that has shown promising clinical efficacy for treatment-resistant depression (Sironi, 2011; Döbrössy et al., 2021). At least 11 brain areas have been studied as candidate targets for relief of treatment-resistant depression by deep-brain stimulation (Drobisz and Damborská, 2019). Particularly effective outcomes have been obtained from electrodes aimed at the medial forebrain bundle (MFB).

In the rat, the MFB is a major, complex, and heterogeneous fiber system that consists of at least 50 components (Nieuwenhuys et al., 1982). Its constituents span a long segment of the neural axis between the basal forebrain and the hindbrain. Debate continues about the structure and appropriate nomenclature of the analogous system in humans and non-human primates (Coenen et al., 2009a,b, 2011, 2012, 2021; Haynes and Haber, 2013; Haber et al., 2021, 2022). In this paper, we will refer to the specific MFB stimulation site that has shown antidepressant efficacy in the deep-brain stimulation studies carried out by Coenen et al. (2020) [MNI (Montreal Neurological Institute)/ACPC (anterior commissure – posterior commissure) system *x* = 6.5 mm, *y* = –2.5 mm (posterior MCP), *z* = –5 mm (below ACPC)] as “MFB.”

Several studies and research teams have shown that bilateral deep-brain stimulation of the MFB causes a strong, immediate, and enduring therapeutic effect in a substantial proportion of patients suffering from treatment-resistant depression (Schlaepfer et al., 2013; Fenoy et al., 2016, 2021; Bewernick et al., 2017; Coenen et al., 2019). Case reports document a swift relapse of depressive symptoms following discontinuation of MFB stimulation and swift remission once stimulation was reinstated (Kilian et al., 2019). However, as in studies focusing on other deep brain stimulation brain targets for relief of depression, prolonged randomized controlled trials on MFB deep brain stimulation have not yet shown clear differences between sham stimulation and deep brain stimulation (Dougherty et al., 2015; Coenen et al., 2019). The putative causes of these failures continue to be debated (Coenen et al., 2019) while reports of successful remediation of symptoms in small cohorts continue to appear (Fenoy et al., 2021).

Preclinical laboratory-animal studies can make use of powerful, invasive methods crucial to linking changes in behavior and psychological processes to the underlying neural circuitry, thereby shedding light on the mechanisms underlying successful clinical interventions. A focus in such research on core psychological processes that have been conserved over the course of mammalian evolution can generate new approaches to intervention, such as development of novel pharmacological agents, behavioral therapies, and interventions such as deep brain stimulation (Coenen et al., 2011; Panksepp and Yovell, 2014; Panksepp, 2016).

In his pioneering work in affective neuroscience, Panksepp proposed a set of highly conserved, “primal emotional systems” (Panksepp and Yovell, 2014). He used the label “SEEKING” to refer to the system mediating investigative behaviors, approach, and “appetitive eagerness:” the highly motivating anticipation of hedonically positive events. He strongly emphasized the anticipatory quality of the emotion generated by activation of the SEEKING system and proposed that its neural substrate differs from that of the coveted hedonic experience.

In Panksepp’s portrayal, the MFB constitutes the core of the SEEKING system. The primary evidence for this is the fact that animals willingly and eagerly turn on electrical stimulation delivered via electrodes arrayed all along the MFB (Panksepp and Yovell, 2014), i.e., they engage in intracranial self-stimulation. Panksepp recognized the neuroanatomical and neurochemical heterogeneity of the MFB, but he and his colleagues ascribed indispensable status to the ascending projections of the midbrain dopamine neurons, which they viewed as energizing the SEEKING system. In that way, the dopamine neurons engage the SEEKING system in intracranial self-stimulation (Ikemoto and Panksepp, 1999), addiction (Alcaro et al., 2007) and relief of depression (Panksepp and Yovell, 2014). Panksepp co-authored several of the early reports documenting the antidepressant effect of MFB stimulation (Coenen et al., 2009b,^2011^, ^2012^), and his portrayal of the SEEKING system provided the initial theoretical foundation for interpreting this effect.

The qualitative approach championed by Panksepp has been paralleled by the development of quantitative methods for measuring reward-seeking behavior, characterizing the underlying neural circuitry, and modeling how the volley of action potentials triggered by MFB stimulation is translated into an enduring record of reward intensity and subsequent pursuit of additional stimulation (Gallistel et al., 1981; Yeomans, 1990; Simmons and Gallistel, 1994; Trujillo-Pisanty et al., 2020). That approach, and the findings and insights it has generated, have yet to be addressed in the literature on the SEEKING system and its role in relief of treatment-resistant depression by deep-brain stimulation. We begin an attempt to fill that lacuna here.

Extension of the quantitative approach to rewarding effects produced by specific optogenetic activation of midbrain dopamine neurons has led to a new view of the circuitry underlying intracranial self-stimulation (Trujillo-Pisanty et al., 2020). On that view, parallel processing channels convey to the behavioral final-common path signals arising in non-dopaminergic MFB fibers and in the ascending projections of midbrain dopamine neurons. We summarize that new view below and explore its potential implications for explaining the relief of treatment-resistant depression by MFB stimulation. Before doing so, we situate the study of intracranial self-stimulation within the context of animal models of depression, we review aspects of depression germane to the question of how MFB stimulation provides relief, and we discuss how research on the effects of such stimulation in rodents could provide insight into the mechanism underlying the antidepressant effect in humans.

### Animal Models of Depression

Behavioral models of depression in laboratory animals often entail exposure to stress followed by measures of consummatory behavior, exploration, disruption of sleep or comfort, and resistance to survival threats (Yan et al., 2010). These measures have been proposed as indices of the putative effectiveness of antidepressant manipulations. Although animal models of depression are widely used, the external and construct validity of such modeling of psychopathology in laboratory animals has been questioned (Molendijk and de Kloet, 2015; Pound and Ritskes-Hoitinga, 2018).

In our view, animals working relentlessly for rewarding stimulation of the MFB and choosing to pursue such stimulation in lieu of competing natural rewards manifest the antithesis of the blunted motivation characterizing depression. Thus, we argue that much may be learned about the core psychological processes underlying depression, their neural substrates, and the therapeutic effect of deep-brain stimulation from research on reward-seeking in laboratory animals in general, and on intracranial self-stimulation in particular. On that view, intracranial self-stimulation provides an animal model amenable to powerful, invasive research methods for investigating psychological processes at the core of depressive symptomatology and for linking these processes to their neural substrates. The processes at the core of the current account are motivational and decisional anhedonia (Zald and Treadway, 2017).

## Depression: Symptoms, Proposed Mechanisms, and Interventions

### Anhedonia in Depression

The most widely used psychiatric diagnostic manual lists anhedonia as one the two cardinal depressive symptoms (American Psychiatric Association, 2013). Originally coined as the complete loss of pleasure (Ribot, 1897), the concept of anhedonia has broadened and differentiated (Treadway et al., 2012; Zald and Treadway, 2017). In contemporary research anhedonia is now operationalized using multiple sub-constructs. Among them are consummatory anhedonia: a reduction in hedonic perception, or enjoyment of rewards (the original definition); motivational anhedonia: a reduced capacity to expend effort in reward pursuit; and decisional anhedonia: an impairment in reward learning and goal selection (Zald and Treadway, 2017).

In studies of anhedonic sub-constructs, deficits in pleasure perception have been distinguished from deficits in motivation and expectation. For example, hedonic ratings of palatable sucrose solutions are not reliably lower in depressed patients than in never-depressed controls (Amsterdam et al., 1987; Berlin et al., 1998). This suggests that systems related to pleasure perception, at least those pertaining to taste and smell, may be unaltered in depression and that the primary deficits do not include consummatory anhedonia. In contrast, motivational and decisional anhedonia are well documented in patients with depression (Cooper et al., 2018). They are less willing to expend effort to acquire rewards of increasing value, and they are less efficient in integrating reward-related information to guide decision making (Treadway et al., 2012; Kumar et al., 2018). Below, we illustrate how intracranial self-stimulation can be used to study motivational and decisional anhedonia. We also emphasize the difficulty of distinguishing anhedonia from other determinants of reward pursuit.

In a Bayesian, decision-theoretic account, depression entails pessimistic expectations about the value of future rewards and possible actions (Huys et al., 2015), an observation well supported by evidence (Cooper et al., 2021). As noted above, Panksepp emphasized the role of the SEEKING system in anticipation of positive outcomes rather than in ongoing hedonic experience, and he viewed hypoactivity of the system as a determinant of depression (Panksepp and Yovell, 2014). That view seems well aligned with the notions of pessimistic expectations and decisional anhedonia. Huys et al. (2015) argue that alterations in model-based, rather than model-free, learning are the most likely route to pessimistic expectations. Definitive isolation of model-based learning in rodents from other forms of learning is not easy to achieve, but it has been demonstrated convincingly (van der Meer et al., 2012; Redish, 2016; Miller et al., 2017). The experimental paradigms in question should be amenable to assessing the effect of MFB stimulation on reward expectations.

It would clearly be of great interest to determine which anhedonia constructs are impacted by MFB stimulation in patients with treatment-resistant depression. Might this be done by comparing appropriate behavioral measures acquired prior to and after the onset of stimulation or a patient-initiated pause (Kilian et al., 2019)?

### Behavioral Activation

The antidepressant efficacy of behavioral activation (Dimidjian et al., 2011) appears to fit well with notions of motivational anhedonia and pessimistic reward expectations. Behavioral activation is a parsimonious psychotherapy that focuses on increasing engagement of depressed patients in reward-seeking activities and decreasing engagement with punishing events. This therapy originated from the hypothesis that systematically increasing engagement in rewarding activities will alleviate depressive symptomatology (Lewinsohn, 1975). Indeed, meta-analyses of research on youth, adult, and elder populations attest to the effectiveness of behavioral activation as a monotherapy for depression (Ekers et al., 2014; Orgeta et al., 2017; Tindall et al., 2017). Moreover, a landmark component analysis study of Cognitive Behavioral Therapy for depression demonstrated that the behavioral activation component of Cognitive Behavioral Therapy is equally as effective at reducing depression symptoms as complete Cognitive Behavioral Therapy (Jacobson et al., 1996).

Although many factors have been proposed as mediators for the antidepressant effect of behavioral activation, a recent systematic review of 21 potential mediators was inconclusive (Dimidjian et al., 2011; Janssen et al., 2021). Consequently, to understand how behavioral activation works, the authors proposed that researchers should turn to the basic behavioral neuroscience of reward seeking (Janssen et al., 2021).

Here, we endorse the idea (Panksepp and Yovell, 2014) that research on intracranial self-stimulation can shed light on the motivational and decisional processes involved in the relief of depression and can contribute to identifying their neural substrates. In particular we consider whether deep-brain stimulation of the MFB achieves antidepressant efficacy by driving one or more of the multiple processes that determine the proclivity of laboratory animals to seek rewarding brain stimulation. Could MFB stimulation and behavioral activation share a common mechanism of action? Recall the evidence that hedonic responses are broadly normal in depressed patients (Amsterdam et al., 1987; Berlin et al., 1998). If so, one would expect that convincing depressed patients to perform activities that re-expose them to pleasurable experiences would correct pessimistic expectations. Could MFB stimulation provide a raised pedestal for expectations, and could this be assessed in an experimental paradigm that isolates model-based learning in rodents (e.g., van der Meer et al., 2012; Redish, 2016; Miller et al., 2017)?

### Psychomotor Stimulants Appear Ineffective as a Monotherapy for Depression

The midbrain dopamine system and its direct afferents have received particular attention in the literature on the antidepressant effect of MFB deep-brain stimulation. Although the authors have been careful to acknowledge the potential contributions of non-dopaminergic neurons, the role of midbrain dopamine neurons occupies center stage in preclinical work inspired by the therapeutic effect of MFB stimulation in humans (Furlanetti et al., 2016; Dobrossy et al., 2019; Döbrössy et al., 2021). On that view, the antidepressant effect of MFB stimulation arises, at least in part, from the *trans*-synaptic activation of midbrain dopamine neurons (Schlaepfer et al., 2013; Döbrössy et al., 2021; Fenoy et al., 2021). This proposal predicts that psychomotor stimulants will have antidepressant effects.

Psychostimulants increase the postsynaptic impact of dopamine by blocking reuptake and/or stimulating release (Kopnisky and Hyman, 2002). The effect of psychostimulants on mood and depression has been under study since the 1930s, predating the discovery of the first and second generation of antidepressants (Hegerl and Hensch, 2017). Early on, researchers concluded that stimulants do not induce reliable antidepressant effects (Hegerl and Hensch, 2017). Those early findings have since received considerable corroboration (Hegerl and Hensch, 2017). Naturalistic studies, randomized controlled trials, reviews, and meta-analyses alike have recorded mixed to negative findings for the effectiveness of several psychostimulants on depression (Candy et al., 2008; Corp et al., 2014; Rohde et al., 2020). Moreover, a pharmaceutical company has scrapped plans to seek regulatory approvals for Lisdexamfetamine (Vyvanse) as adjunct treatment for depression after two large, multi-center, stage three randomized controlled trials failed to demonstrate a clinical effect (Hegerl and Hensch, 2017).

It has been proposed that interest in the antidepressant efficacy of psychostimulants persists due to the induction of a fast-acting, but short-lived, mood elevation (Candy et al., 2008; Malhi et al., 2016). This suggests that stimulants influence mood differently than established antidepressants, which have a delayed clinical onset of days or weeks (Malhi et al., 2016; Harmer et al., 2017). Given that the mood elevation produced by psychostimulants is typically short lived, one may wonder whether such drugs can induce a lasting mood improvement when their bioavailability is increased. An initial answer is provided by a randomized controlled trial carried out to assess the effectiveness of an extended-release formulation of methylphenidate as an adjunct medication for treatment-resistant depression (Patkar et al., 2006). No clinical efficacy was found. Further research is needed to evaluate whether the rapid-onset mood elevation inducted by psychostimulants can become sustained by drug formulation or dose regimen. In addition, it would be of interest to assess the efficacy of drugs that target the dopamine transporter more specifically than conventional psychomotor stimulants. At present, the prescription of stimulants for depression remains controversial: Clinicians are advised to use stimulants sparingly and only as additions to other antidepressant drugs for the purpose of improving arousal and tiredness (Malhi et al., 2016).

The lack of robust evidence that psychomotor stimulants are effective in relief of depression raises concerns about the attribution of the strong antidepressant effect of MFB stimulation to the indirect activation of midbrain dopamine neurons. Further research on the possible effect of dopamine agonists on depression could focus on whether these drugs exert influence on motivational and decision-making anhedonia in depressed individuals. Optogenetic methods (Yizhar et al., 2011) provide a powerful way to assess the influence of enhanced dopamine tone on reward pursuit and reward expectations in rodents. We touch on that issue in the following section, in which we discuss the rewarding effect of MFB stimulation in laboratory animals. We highlight evolving views of the role played by midbrain dopamine neurons, and we entertain the possibility that the antidepressant effect of MFB stimulation in humans may involve non-dopaminergic components of brain reward circuitry.

## Intracranial Self-Stimulation of the Medial Forebrain Bundle

### Overview

The study of brain reward circuitry was launched by Olds and Milner’s (1954) discovery of electrical, intracranial self-stimulation. Olds noticed that a rat returned repeatedly to a location in an open field where it had previously received deep-brain stimulation (Olds, 1973). An apparatus was quickly constructed to allow the rat to trigger the stimulation (Milner, 1989). The experimenters then observed a gripping spectacle: the rat worked energetically and persistently for the electrical reward. The location of the electrode tip was not verified definitively, but x-ray imaging suggested that the tip was located in or near the septal area (Milner, 1989), an important source of MFB fiber (Nieuwenhuys et al., 1982).

A flood of research findings emerged during the first decade following the seminal discovery of Olds and Milner. Among these were the results of mapping studies that documented particularly vigorous lever-pressing behavior for stimulation of the MFB (Olds and Olds, 1963). That decade also saw the introduction of pharmacological approaches (Olds, 1958b; Stein and Ray, 1960; Stein and Seifter, 1961). Refinement of behavioral methods for drawing neurochemical inferences about the reward substrate and development of increasingly specific pharmacological agents helped build a consensus that dopamine neurons play a crucial role in the phenomenon (Franklin, 1978; Wise, 1978, 1980). In parallel, psychophysical inference of anatomical and physiological properties of the directly activated neurons underlying the rewarding effect implicated non-dopaminergic neurons with highly excitable (Yeomans, 1975, 1979), myelinated (Shizgal et al., 1980; Gallistel et al., 1981; Bielajew and Shizgal, 1982, 1986) axons that course through the MFB. The properties of these neurons contrast sharply with those of dopaminergic MFB axons, which have high thresholds to activation by extracellular electrical currents (Guyenet and Aghajanian, 1978; Yeomans et al., 1988; Anderson et al., 1996). To resolve these discrepancies, the “series-circuit” hypothesis portrays the myelinated MFB axons as a source of direct or indirect synaptic input to midbrain dopamine neurons whose excitation is responsible for the rewarding effect (Shizgal et al., 1980; Wise, 1980; Bielajew and Shizgal, 1986). The discovery that rodents also work vigorously for specific, optical excitation of opsin-expressing midbrain dopamine neurons (Adamantidis et al., 2011; Witten et al., 2011; Kim et al., 2012) appeared to fit the series-circuit hypothesis neatly: On that view, the optical input achieves directly what the electrical stimulation achieves indirectly by driving mono- or multi-synaptic inputs to midbrain dopamine neurons.

Despite its face validity, the series-circuit hypothesis has been challenged by recent findings (Trujillo-Pisanty et al., 2020) obtained by measurement of operant performance as a function of both the strength and cost of the reward. Blockade of the dopamine transporter enhanced the reward-seeking behavior, but it did so differently in the cases of electrical and optical self-stimulation, thus violating predictions of the series-circuit hypothesis. To account for both datasets, a new architecture for brain reward circuitry was proposed. In this new model, the myelinated MFB axons and the axons of the midbrain dopamine neurons give rise to reward signals that converge, via separate routes, on the behavioral final-common path for the evaluation and pursuit of rewards.

In the following subsections we summarize evidence that gave rise to the series-circuit hypothesis as well as evidence that challenges this longstanding account of brain-reward circuitry. We then discuss the implications of the convergence model for interpretation of the effect of MFB stimulation on relief of treatment-resistant depression.

### Intracranial Self-Stimulation of the Medial Forebrain Bundle: Phenomenology

Rats and other laboratory animals manifest exceptionally strong motivation to earn rewarding MFB stimulation. To gain access to a lever that administers strong MFB stimulation, rats will run uphill leaping over hurdles (Edmonds and Gallistel, 1974) or endure foot-shocks administered by an electrified grid (Olds, 1958b). Provided with continuous access to rewarding MFB stimulation, rats may lever press for 24 h or more until they drop from exhaustion (Olds, 1958a). In an unpublished account (Gardner, Eliot, *personal communication*), macaques refused to surrender a manipulandum that triggered rewarding electrical stimulation of the MFB. At the conclusion of the test session, the experimenter was unable to muster sufficient strength to pry the device from the monkey’s grip and had to wait patiently for the animal to relent. The problem was solved by a mechanic on the air-force base where the experiment was conducted. He rigged a powerful motor normally used to retract the landing gear of an airplane to pull the manipulandum away from the monkey.

The extraordinary zeal, vigor, and persistence shown by laboratory animals working for rewarding MFB stimulation provides a diametrically opposed image of the weakened motivation and goal seeking shown by patients with depression. In the throes of a depressive episode, even goals that normally loom as urgent can lose their incentive power. Could hypoactivity of conserved neural circuitry subserving electrical self-stimulation in laboratory animals account for the motivational deficit burdening depressed humans? If so, it seems plausible that chronic electrical stimulation of such pathways could provide relief and that a deep understanding of the neural mechanisms underlying electrical self-stimulation could contribute further to the development of novel, effective treatments.

### Contingency

In intracranial self-stimulation experiments, delivery of stimulation is contingent upon the behavior of the subject. In contrast, deep-brain stimulation of the MFB for relief of treatment-resistant depression is delivered non-contingently and typically, continuously (but see Scangos et al., 2021). Does this pose an insurmountable problem for efforts to relate these two applications of MFB stimulation? We address the issue of contingency below within the framework of the convergence model. Here, we point out that an effect of non-contingent MFB stimulation has long been prominent in the literature on intracranial self-stimulation.

Non-contingent delivery of free stimulation trains prior to a trial increases the vigor of subsequent stimulation-seeking behavior, a phenomenon called the “priming effect (Gallistel, 1966; Edmonds and Gallistel, 1974).” Such non-contingent pretrial stimulation also exerts a powerful influence on reward selection. When a long delay intervened between delivery of non-contingent. pretrial stimulation, thirsty rats chose an arm of a T-maze that led to water, whereas after zero or short delays, they chose an alternate arm that led to a goal box in which rewarding stimulation was delivered (Deutsch et al., 1964). Such energizing and directing effects are the two defining characteristics of motivation. That they can arise following non-contingent delivery of stimulation provides a conceptual link between the intracranial self-stimulation phenomenon and the hypothesis that deep-brain stimulation of the MFB may offset motivational anhedonia. That said, the priming effect of MFB stimulation can be construed as a rapidly decaying aftereffect of exposure to strong, episodic rewards (Sax and Gallistel, 1991). If the priming effect is to be linked convincingly to the antidepressant action of MFB stimulation in humans, it must be demonstrated that continuous non-contingent stimulation can exert a motivational influence on self-stimulation performance. Below, we discuss how such an experiment could be done.

### Measurement of Electrical Intracranial Self-Stimulation

Before we can explore more deeply how research on intracranial self-stimulation can inform our understanding of the mechanism by which deep brain stimulation of the MFB relieves treatment-resistant depression, we need to delve into how ICSS is measured and how conclusions about mechanisms are drawn from the behavioral observations. What do changes in the observed performance of the animal reveal about the internal variables that control goal-directed behavior and its neural underpinnings?

Experimenters adopted a simplistic “more is better” approach to the measurement of ICSS in early studies: manipulations that increased response rates were deemed, implicitly or explicitly, to have boosted the rewarding effect of the stimulation (Olds et al., 1956; Crow, 1970). Among the obstacles on which this approach founders is the sigmoidal form of the curves that relate response rates to stimulation strength. There is a ceiling on response rate, and responding will cease when stimulation strength falls too low. Thus, the magnitude of any change in response rate will depend on the level observed in the control condition, which will vary due to the scatter of stimulation sites. Moreover, response rates are subject to multiple influences in addition to the strength of the rewarding effect (Hodos and Valenstein, 1962). For example, how can overall suppression or enhancement of response rates for fixed stimulation parameters be distinguished from changes in reward?

Measurement of response rates for a fixed set of stimulation parameters has been largely replaced by the “curve-shift” method (Barry et al., 1974; Yeomans, 1975; Edmonds and Gallistel, 1977; Miliaressis et al., 1986). A measure of response vigor, such as response rate, is obtained at each of a set of stimulation strengths that drive the response variable through its full range. This traces the full psychometric curve. The effect of a manipulation, such as the administration of a drug, is assessed by whether and how it displaces the sigmoidal psychometric curve along the axis representing stimulation strength, typically the pulse frequency. The “more is better” approach represented by the measurement of changes in the rate of responding for a fixed set of stimulation parameters is thus replaced by a “bang for the buck” approach: Manipulations that boost the effectiveness of the rewarding stimulation reduce the pulse frequency required to produce responding of a particular vigor (the response criterion), whereas manipulations that reduce rewarding effectiveness produce the opposite effect, necessitating a compensatory increase in pulse frequency in order to restore responding to its initial level. The sigmoidal curves are roughly parallel when plotted against the logarithm of the stimulation strength. Thus, the magnitude of the observed shift is independent of the response criterion.

Proponents of the curve-shift method have argued that it removes the ambiguity inherent in interpretation of changes in response-rate measures (Carlezon and Chartoff, 2007). On that view, lateral displacements of the psychometric curve reflect changes in reward effectiveness, whereas changes in the vertical scaling of the curve reflect changes in motoric capacity. Alas, that hopeful formulation is not well supported by evidence: adding weight to the lever produces both vertical rescaling and lateral shifts (Frank and Williams, 1985; Fouriezos et al., 1990). The reason for this is intuitive: the vigor of performance depends both on the *cost* of the reward as well as on its strength. To address this, Shizgal and colleagues measured performance while varying both strength and cost (Arvanitogiannis and Shizgal, 2008; Hernandez et al., 2010; Breton et al., 2013).

**FIGURE 1:**
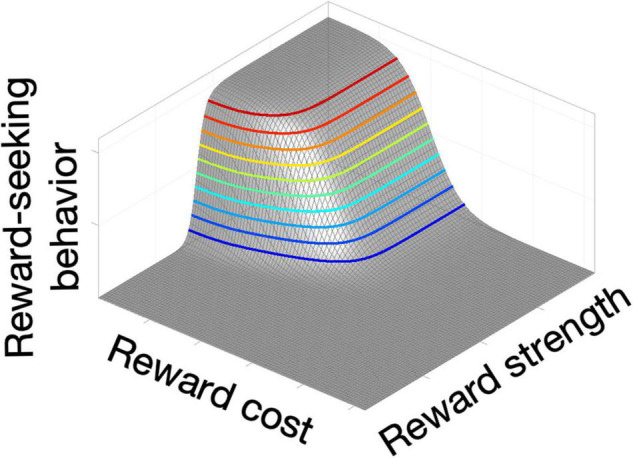
Schematic of the reward mountain, a method used to measure reward seeking while varying both reward strength and cost.

Time weighs heavily in accounts of foraging behavior and conditioning (Gallistel and Gibbon, 2000). A reward that can be secured rapidly outweighs one that is delivered only following a prolonged behavioral investment (Solomon et al., 2017). The former is said to have a lower “opportunity cost” than the later. Shizgal and his team manipulate opportunity costs in ICSS experiments by requiring rats to put time on a clock: the rats do so by holding down a lever until the accumulated time meets the experimenter-imposed criterion for earning a stimulation train (Breton et al., 2009). They quantify the rat’s behavior by measuring the partitioning of the rat’s time between “work” (holding down the lever) and “leisure,” anything else the rat chooses to do, such as resting, grooming or exploring. Not surprisingly, the proportion of the rat’s time devoted to working for a reward of a given strength (“time allocation”) declines as the required opportunity cost grows. Conversely, time allocation grows as a function of stimulation strength (e.g., pulse frequency) when opportunity cost is held constant. By measuring time allocation over a large set of opportunity costs and pulse frequencies, a fitted surface is obtained that looks like the corner of a plateau: the “reward mountain” (Arvanitogiannis and Shizgal, 2008; Figure 1).

The reward-mountain method removes a key source of ambiguity inherent in curve-shift measurements. Response-rate-versus-pulse-frequency curves are displaced laterally either by altering reward strength or the effort required to press the lever. In contrast, the reward mountain is displaced in orthogonal directions by manipulation of the strength and cost variables. This disambiguation is crucial for interpreting displacement of the reward mountain by experimental variables. As we will describe shortly, application of the reward-mountain method has falsified the long-standing “series-circuit” model of brain reward circuitry and has inspired its replacement with a new candidate germane to interpreting the effects of deep-brain stimulation in humans: the convergence model.

### Mapping the Reward-Mountain Model Onto Stages of Neural Processing

**FIGURE 2:**
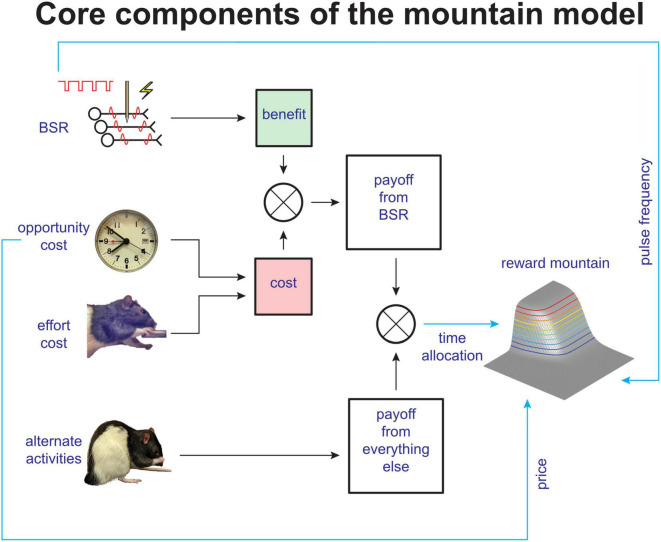
Simplified schematic of core components of the reward-mountain model (from Trujillo-Pisanty et al., 2020).

The interpretation of shifts in the position of the reward-mountain is based on a quantitative model of how the volley of action potentials triggered by the stimulation is translated into observable operant performance (summarized qualitatively in Figure 2). The formal derivation is provided in the supporting information for Trujillo-Pisanty et al. (2020). An alternative formulation, derived from reinforcement-learning principles, has been developed by Niyogi et al. (2013, 2014).

The top row of Figure 2 depicts the translation of the physical parameters of a stimulation train into a reward-intensity value stored in memory (nicknamed “benefit”). The “reward-growth” function that does the heavy lifting in this regard has been measured by Mark and Gallistel (1993); Simmons and Gallistel (1994), and Leon and Gallistel (1998) using operant matching on concurrent variable-interval schedules. The reward-mountain method can distinguish between effects of drugs and other manipulations that operate on the input to the reward-growth function (the green box labeled “benefit” in Figure 2) and those arising from all subsequent stages. Modulation of the input to the reward-growth function shifts the reward mountain along the pulse-frequency axis. This is intuitive given the long-standing view that the input is the aggregate rate of firing in the neurons subserving the rewarding effect (the total action-potential count elicited by a pulse train of a given duration) (Gallistel, 1978; Gallistel et al., 1981; Simmons and Gallistel, 1994). If the number of stimulated neurons is increased by boosting the stimulation current, a compensatory decrease in pulse frequency will be required to hold the aggregate firing rate constant, thus shifting the reward mountain along the pulse-frequency axis. This has been confirmed experimentally (Arvanitogiannis and Shizgal, 2008).

The neural signals responsible for the rewarding effect of electrical or optical stimulation arise initially as a volley of action potentials in neurons adjacent to the tip of an electrode or fiber-optic probe. Identifying these neurons and tracing their outputs must lead to the circuitry that translates the stimulation-induced volley into an enduring record of reward intensity. The reward-mountain model and the associated measurement method tell us whether a manipulation such as administration of a drug or delivery of constant background stimulation alters reward processing prior to or beyond the reward-computing and encoding circuitry.

Figure 2 shows that multiple variables intervene between the output of the reward-growth function (the green box labeled “benefit”) and the observable behavior of the rat. These variables, which all shift the mountain along the cost axis (Breton et al., 2013, 2014; Trujillo-Pisanty et al., 2020), include the subjective effort entailed in holding down the lever, the value of alternate activities, and a scale factor applied to the output of the reward-growth function (not shown). Thus, although the reward-mountain method reduces an important source of ambiguity in the interpretation of curve-shift data, we must put some water in our wine. Other sources of ambiguity persist in the interpretation of data obtained by means of the reward-mountain method, and they are likely to do so until the conceptual entities in the model are replaced by measurable neural signals in identified neurons.

To our knowledge, only two studies have evaluated the effect of continuous background stimulation of the MFB on intracranial self-stimulation (Walker and Fouriezos, 1995; Rea et al., 2014). Neither employed the reward-mountain method, and in the study by Rea et al. (2014) background stimulation was not delivered while the rats were working for the reward. Given the effectiveness of continuous MFB stimulation in relieving treatment-resistant depression, it would be highly worthwhile to use the reward-mountain method to revisit the question of whether and how continuous background stimulation of the MFB alters pursuit of additional stimulation. If there is an effect of such background stimulation, is it brought to bear on the input or output side of the reward-growth function (or both)? An effect on the output side (i.e., a rightwards shift of the reward mountain along the cost axis) would be compatible with the concepts of motivational and decisional anhedonia. For example, such an effect could arise from summation between the tonic effect of the continuous stimulation (see the full, updated convergence model in Supplementary Figure 1) with reward-intensity values retrieved from memory. Such summation would offset pessimistic reward expectations in depressed individuals (Sherdell et al., 2012; Huys et al., 2015), thus increasing the proclivity to invest effort in reward pursuit. That proclivity would also be boosted by a reduction in subjective effort costs, another of the perturbations that can shift the reward mountain rightwards along the cost axis.

Let us now consider how the reward-mountain model recasts the role of midbrain dopamine neurons in intracranial stimulation, and, potentially, in the relief of treatment-resistant depression by MFB stimulation.

### Dependence of Intracranial Self-Stimulation of the Medial Forebrain Bundle on Dopaminergic Neurotransmission

Drugs that alter dopaminergic neurotransmission produce systematic changes in rate-frequency curves obtained from self-stimulating rats (Franklin, 1978; Wise and Rompré, 1989; Wise, 1996). A particularly elegant demonstration was provided by Gallistel and Karras (1984) in rats working for electrical stimulation of the MFB. The curves were driven leftwards by 2 mg/kg of amphetamine (which increases dopamine release) and rightwards by 0.3 mg/kg of pimozide (which blocks the dopamine D2 and 5HT-7 receptors); the effects of the two drugs canceled when administered together.

We have shown (Hernandez et al., 2010; Trujillo-Pisanty et al., 2020) that drug-induced shifts in rate-frequency curves could arise either from drug-induced modulation of the input to the reward-growth function or from modulation of its output. This ambiguity in the interpretation of curve shifts induced by changes in dopaminergic neurotransmission is resolved by application of the reward-mountain method. In 7/10 rats treated with the specific dopamine transporter blocker, GBR12909, the reward mountain measured in rats working for electrical MFB stimulation was shifted reliably along the opportunity-cost (“price”) axis, whereas no rat demonstrated a reliable shift along the pulse-frequency axis (Hernandez et al., 2012). The dopamine D2/5HT-7 receptor blocker, pimozide, shifted the reward mountain reliably along the price axis in 5/6 rats, whereas no rat demonstrated a reliable shift along the pulse-frequency axis (Trujillo-Pisanty et al., 2014). Thus, these studies show that the drug-induced modulation of dopaminergic neurotransmission altered reward seeking by means of actions at or beyond the output of the reward-growth function.

### Convergent Causation: Multiple Determinants of Reward Seeking

The results obtained in pharmacological studies employing the reward-mountain method send an important message beyond the role of neurotransmitter systems in reward seeking. These results remind us that we ignore convergent causation (“equifinality”) at our peril. It is obvious that a given measurement, such as a change in response vigor, may arise from multiple causes, but the ease with which convergent causation can be obscured and ignored in the interpretation of curve shifts is often unappreciated.

**FIGURE 3:**
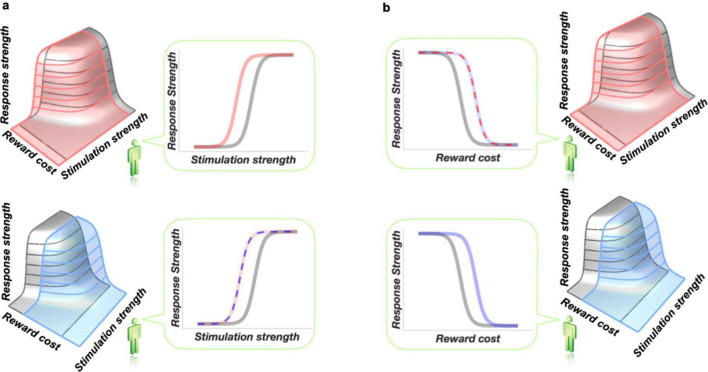
The inherent ambiguity of two-dimensional scaling of operant-conditioning data, such as rate-frequency and progressive-ratio curves. Redrawn from Hernandez et al. (2010). The two-dimensional graphs in panel **(a)** are drawn from the perspective of the little green figure, who views the three-dimensional structure from the stimulation-strength axis. The two-dimensional graphs in panel **(b)** are also drawn from the perspective of the little green figure, but here, this observer views the three-dimensional structure from the reward-cost axis. For a video illustrating this issue in more detail, see: https://spectrum.library.concordia.ca/978205/. The little green figure is from Shutterstock Images LLC.

In conventional curve-shift studies of the effects of drugs on intracranial self-stimulation, the data are typically plotted in two dimensions, with the stimulation-strength variable (usually pulse frequency) on the *x*-axis and a response-strength measure (usually response rate) on the *y*-axis, as shown in the top-right panel of Figure 3a. Implicitly, the independent variable, plotted on the *x*-axis, is taken as the *cause* of the variation observed in the dependent variable, plotted on the y-axis. We assume, quite reasonably, that boosting the strength of the stimulation (by raising the frequency or current) will increase the intensity of the resulting rewarding effect. In the baseline condition (gray), this attribution is fine: we are confident that it is only the stimulation strength that varies from trial to trial. A problem arises when we introduce a second independent variable: administration of a drug. The effect of stimulation strength on reward intensity is salient on our minds when we view the graph. Thus, when we then observe a drug-induced curve-shift (top-right panel of Figure 3a), we are prone to assuming that this is due to a drug-induced change in sensitivity to stimulation strength: the variable represented on the *x*-axis. Such a shift would displace the reward mountain leftwards along the pulse-frequency axis (top-left panel of Figure 3a). In such a case, the two-dimensional graph correctly captures the shift, and the observer viewing that graph intuits the correct conclusion.

The lower-right panel of Figure 3a shows that the convert assumption of a leftward shift of the reward-growth function is premature. The very same shift in the rate-frequency curve could arise from a shift of the mountain along the cost axis (lower-left panel of Figure 3a; thin, blue, dashed curve in the lower-right panel). In such a case, the intuitive conclusion (thick, solid, pink curve in the lower-right panel) drawn by an observer focused on the *x*-axis label is incorrect. This is indeed the case in intracranial self-stimulation experiments entailing manipulation of dopaminergic (Hernandez et al., 2012; Trujillo-Pisanty et al., 2014) or cannabinoid (Trujillo-Pisanty et al., 2011) signaling. In those experiments, the reward mountain shifts along the cost axis (e.g., bottom-left panel of Figure 3a), which does not appear in the two-dimensional graph, and not along the stimulation-strength axis that is so salient in the mind of the observer. The intracranial self-stimulation data are consistent with the thin, dashed, blue curve in the lower-right panel, and not with the thick, solid, pink curve representing the intuitive conclusions drawn by experimenters who used the curve-shift method. The trap that causes intuitive interpretation of rate-frequency curves to go awry is illustrated in more detail in a video available here: https://spectrum.library.concordia.ca/978205/.

Analogous ambiguity is inherent in interpretation of progressive-ratio data. In progressive-ratio experiments (Hodos, 1961), response strength is plotted against the effort cost of the reward, as determined by the required number of lever presses. That scenario is depicted in Figure 3b. In the baseline condition (gray curve in top-left panel), there is a clear and systematic relationship between response strength and the reward cost. The observer viewing the two-dimensional data (left column) thus tends to jump to the premature conclusion that a drug-induced shift in a response-rate-versus-fixed-ratio curve is due to a change in sensitivity to reward cost: the variable plotted on the *x*-axis. That interpretation (thick, solid blue curve in the top-left panel of Figure 3b) would be correct if the reward mountain indeed moved in the direction shown in the lower-right panel, but it would be incorrect if the shift were in the orthogonal direction (thin, dashed, pink curve in the upper-left panel). When plotted in two-dimensions (left column), changes in sensitivity to either reward strength or reward cost can produce indistinguishable results (superimposed curves in the upper-left graph), but only sensitivity to reward cost is salient in the mind of the viewer. The three-dimensional representation (right column) made possible by the reward-mountain methods resolves the ambiguity and makes both the strength and cost axes salient.

Like the reward-mountain method, the effort-expenditure-for-rewards-task developed for use in experiments with human participants measures reward pursuit as a function of both the cost and strength of reward (Treadway et al., 2009). Thus, this task could achieve the same distinction as the reward-mountain method between variables acting at, or prior, to the input of the reward-growth function and variables acting at, or beyond, its output. However, such a distinction is possible only when the direction in which the mountain surface can be determined. A non-linear reward-growth function is required. The use of small monetary rewards may well fail to provide the required non-linearity. We speculate that the required non-linearity would be achieved if a reward that had to be consumed in the laboratory at the end of the session were substituted for the monetary payoffs. For example, a chocolate lover could be informed that they had the opportunity to earn various amounts of their preferred variety, with the proviso that they had to consume it within a given time period at the end of the session. The reward-growth function for such a payoff will saturate because the participant will know that amounts beyond a given mass will exceed what could reasonably be consumed and enjoyed within the available time.

Figure 3 illustrates how easy it is to prematurely adopt one of a set of convergent causes as the explanation for an observation and to ignore less-salient alternatives. As the figure shows, the reward-mountain method is indeed able to distinguish one set of potential causes, those acting at the input to the reward-growth function, from a second set, those acting at or beyond the output. However, Figure 2 counsels caution. It shows that the mountain method fails to distinguish between the members of the second set of potential causes: multiple variables that can shift the mountain along the price axis, including subjective estimates of opportunity costs, effort costs, and the value of alternate activities. Although we have established that at least one member of that second set depends on dopaminergic signaling (Hernandez et al., 2012; Trujillo-Pisanty et al., 2014), that does not prove that all the other members do as well. Thus, it would be unwise and unwarranted to leap to the premature conclusion that dopaminergic mechanisms underlie all shifts of the mountain along the price axis.

Convergent causation is no less germane to the interpretation of antidepressant effects of MFB stimulation in humans. Let us keep that in mind when we later address the putative role of dopamine signaling in the antidepressant effect of MFB stimulation.

### Dependence of Intracranial Self-Stimulation of the Medial Forebrain Bundle on Direct Activation of Myelinated Descending Fibers

In parallel with the initial work that established the dependence of MFB self-stimulation on dopaminergic neurotransmission, detailed psychophysical studies were carried out to characterize the directly stimulated neurons responsible for the rewarding effect (Gallistel et al., 1981). The estimated characteristics include recovery from refractoriness (Yeomans, 1975, 1979; Bielajew et al., 1982), conduction velocity (Shizgal et al., 1980; Bielajew and Shizgal, 1982, 1986; Murray and Shizgal, 1996a,b), frequency following (Gallistel, 1978; Simmons and Gallistel, 1994; Solomon et al., 2015), and the behaviorally relevant direction of conduction (Bielajew and Shizgal, 1986). The results are consistent with the hypothesis that the principal constituents of the directly activated substrate for MFB self-stimulation are neurons with descending myelinated axons. In contrast, the dopaminergic fibers in the rat MFB have slow-conducting (Feltz and Albe-Fessard, 1972; Takigawa and Mogenson, 1977; Guyenet and Aghajanian, 1978; German et al., 1980; Maeda and Mogenson, 1980; Yim and Mogenson, 1980), unmyelinated (Hattori et al., 1991) axons with relatively long refractory periods (Anderson et al., 1996) that ascend from the midbrain to the forebrain (Ungerstedt, 1971). The series-circuit hypothesis (Shizgal et al., 1980; Wise, 1980; Bielajew and Shizgal, 1986) was proposed to reconcile the pharmacological data implicating dopaminergic neurons in MFB self-stimulation with the portrayal that has emerged from the psychophysical studies.

### The Series-Circuit Model of Intracranial Self-Stimulation

**FIGURE 4:**
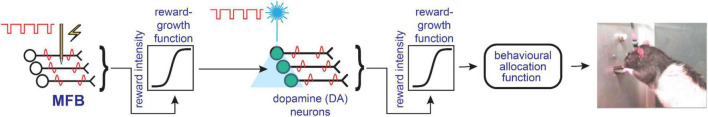
Simplified schematic depicting the basic components of the series-circuit model of brain reward circuitry, redrawn from Trujillo-Pisanty et al. (2020).

The series-circuit model attempts to accommodate both the psychophysical and pharmacological data by concatenating two sets of neurons. In that model, MFB-projecting neurons with myelinated axons dominate the directly stimulated stage. They do so because they are much more readily excited by electrical currents than the fine dopaminergic axons in the MFB, which have high threshold to electrical activation (Guyenet and Aghajanian, 1978; Yeomans et al., 1988; Anderson et al., 1996). Instead of being driven directly by MFB electrodes, the series-circuit model posits that midbrain dopamine neurons are excited mono- or poly-synaptically by input from the directly activated, myelinated fibers. Figure 4 provides a simplified sketch of this hypothesis. As explained above, the failure of the reward mountain to shift along the pulse-frequency axis following blockade of the dopamine transporter or dopamine receptors implies that the drugs acted beyond the output of the reward-growth function. Thus, a reward-growth function is positioned in Figure 4 between the output of the directly activated MFB neurons and the midbrain dopamine cells. The second reward-growth function (beyond the output of the dopamine neurons) is required to accommodate data from an experiment in which rats worked for specific, optical stimulation of midbrain dopamine neurons (Trujillo-Pisanty et al., 2020).

### Evidence Inconsistent With the Series-Circuit Model

The discovery that rodents will work for specific optogenetic stimulation of midbrain dopamine cells seems to fit the series-circuit model nicely. However, application of the reward-mountain method to optical self-stimulation places a seemingly insurmountable obstacle in the path of the series-circuit model.

Trujillo-Pisanty et al. (2020) used the reward-mountain method to test the effect of dopamine-transporter blockade on reward-mountain measurements obtained from rats working for direct, specific optogenetic activation of midbrain dopamines. As in the case of electrical self-stimulation, the rewarding effect (particularly following administration of the specific dopamine transporter blocker, GBR-12909) started to saturate at pulse frequencies well within the frequency-following capabilities of the dopamine neurons. This implies that a saturating reward-growth function is positioned downstream of the activated dopamine neurons (Figure 4). In contrast to the results obtained with the same drug on electrical self-stimulation of the MFB, they found that the mountain was shifted leftwards along the pulse-frequency axis by dopamine transporter blockade. That result implies that the rewarding effect was boosted by an action at or before the *input* to the reward-growth function. This finding refutes the series-circuit model, because positioning a reward-growth function downstream from the dopamine neurons predicts that perturbation of dopaminergic neurotransmission would also shift of the reward mountain along the pulse-frequency axis in rats working for electrical MFB stimulation, whereas Hernandez et al. (2012) and Trujillo-Pisanty et al. (2014) showed that it does not. In order to accommodate both sets of results, Trujillo-Pisanty et al. (2020) proposed a new architecture: the convergence model.

### The Convergence Model

**FIGURE 5:**
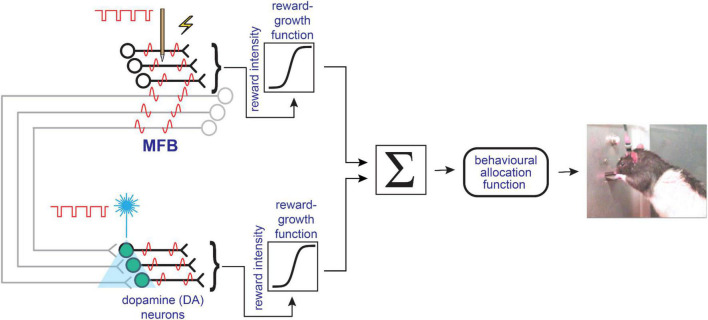
Simplified depiction of the convergence model, redrawn from Trujillo-Pisanty et al. (2020). See Supplementary Figure 1 for an updated version of the full model.

A simplified summary of the convergence model is provided in Figure 5. The full model and extensive computer simulations supporting it are provided in the report by Trujillo-Pisanty et al. (2020). Supplementary Figure 1 provides an update to this full model to address potential effects of continuous MFB stimulation. In this architecture, the myelinated MFB axons and the midbrain dopamine neurons have parallel access to the final common path for reward pursuit. The convergence model thus elevates the state of the myelinated pathway. In the series-circuit model, the stimulated MFB axons are merely one of many sets of inputs to the midbrain dopamine neurons (Watabe-Uchida et al., 2012), which are the gatekeepers to the final common path for reward estimation and pursuit. In contrast, the convergence model gives the myelinated pathway an independent voice in the chorus vying for control over the behavioral final common path, one that can dominate under the conditions of electrical self-stimulation experiments.

The convergence model accommodates a number of prior findings that fit poorly with the series-circuit model. These include the results of studies employing radical ablation methods that eliminated most of the forebrain terminations of ascending dopamine neurons (Huston and Borbély, 1973; Pritzel et al., 1983), a study of the effect of cytotoxic lesions of the nucleus-accumbens terminal field (Johnson and Stellar, 1994), and a comparison between frequency following in midbrain dopamine neurons and the substrate for the rewarding effect of electrical MFB stimulation (Cossette et al., 2016).

Research on the role of dopaminergic neurons in reward seeking has accomplished so much and achieved such prominence as to overshadow the established and potential contributions of other neural populations. The ascending dopaminergic projection from the midbrain is merely one of over 50 distinguishable components of the MFB (Nieuwenhuys et al., 1982). Which of the others contribute to the evaluation and pursuit of rewards and in what ways? The convergence model encourages us to give greater consideration to the non-dopaminergic components, which include descending projections that pass through or near the midbrain region housing dopamine cell bodies and continue deeply into the brainstem (Nauta et al., 1982).

Rompré and Miliaressis (1985, 1987) have described an array of electrical self-stimulation sites that runs longitudinally along the core of the mesencephalon, well caudal to the dopamine cells bodies clustered in the ventral tegmental area (VTA). Boye and Rompré (1996) demonstrated that axons contributing to the rewarding effect directly link sites in this mesencephalic array with the VTA and lateral hypothalamus. Although the behaviorally relevant direction of conduction in these fibers is unknown, the finding of Boye and Rompré could arise from reward-related MFB projections that pass through the VTA en route to more caudal regions. Such projections would be suitable candidates for the long-sought descending path and for the channel in the convergence model that parallels the midbrain dopamine neurons en route to the behavioral final common path for reward seeking. Indeed, Huston (1982) has long interpreted the results of radical ablation experiments carried out by his team (e.g., Huston and Borbély, 1973; Pritzel et al., 1983) to imply that the critical circuitry underlying self-stimulation, and reinforcement of operant behavior more generally, lies in the deep brainstem.

### Finding the Parallel Path

Most of the research that gave rise to the series-circuit model was carried out in the 1970s and 1980s. The reader may well ask why the neurons with myelinated axons implicated by this work as the directly stimulated substrate for MFB self-stimulation have not since been found. In our view, this is due principally to the nature of the tools that were available until recently for anatomical tracing and for determining the necessity and causal role of identified neural pathways in behavioral effects of brain stimulation. Painstaking manual methods, typically applied to small numbers of selected tracer-injection sites, were long required to trace axonal trajectories between their cell-body origins and postsynaptic targets (Cowan et al., 1972; Veening et al., 1982; Wouterlood et al., 2014). Applying such methods required prior knowledge of the origin and/or termination of a given pathway. In the absence of such prior information, researchers studying effects of electrical brain stimulation had few options for tracing axons coursing past the stimulation site (e.g., Fink and Heimer, 1967; Honig and Hume, 1989).

An array of recently developed trajectory-tracing technologies greatly enhance feasibility, accuracy, specificity, and speed (Wouterlood et al., 2014; Lanciego and Wouterlood, 2020). Tissue-clearing methods (Tian et al., 2020; Ueda et al., 2020; Richardson et al., 2021) render entire rodent brains optically transparent. Via stochastic electrotransport (Kim et al., 2015), fluorescent antibodies can be driven efficiently into cleared rodent brains to label specific neural populations. Via light-sheet microscopy (Mano et al., 2018; Migliori et al., 2018; Chakraborty et al., 2019; Hillman et al., 2019), the cleared tissue can be sectioned optically, thus eliminating registration issues and the need for manual manipulation of delicate tissue sections. Segmentation and image-analysis software makes it possible to trace single axons throughout the labeled, cleared brain (Berger et al., 2018; Gao et al., 2019). Achieving this for individual myelinated axons, such as those coursing through MFB self-stimulation sites, is no longer a dream. (For an example of long-distance tracing of fluorescently labeled myelinated axons in cleared tissue, see Gao et al., 2019). The new methods not only provide detailed information about connections (origins and terminations of neural projections), they also trace trajectories, which is crucial to identifying the directly activated neurons subserving behavioral effects of deep-brain stimulation.

Once the trajectories of the axons of interest have been traced, optogenetic methods (Yizhar et al., 2011) can render the neurons that give rise to particular MFB components optically excitable, thereby making it possible to determine whether driving these cells produces rewarding and/or motivating effects. Identification of the terminal fields of the MFB-projecting neurons, coupled with optogenetic silencing methods (Yizhar et al., 2011; Wiegert et al., 2017) provide complementary means for assessing the necessity of these neurons for the rewarding effect of MFB stimulation. By recording the activity of these neurons in response to rewarding MFB stimulation, it can be determined whether the properties of their axons correspond to the psychophysically derived portrait of the fibers subserving MFB self-stimulation (e.g., Rompré and Shizgal, 1986; Shizgal et al., 1989; Murray and Shizgal, 1996b; Cossette et al., 2016).

## Implications for Research on the Antidepressant Effect of Deep-Brain Stimulation

### Stimulation Parameters

Before we discuss the implications of the convergence model for research on the antidepressant effect of deep-brain stimulation, it is important to address the issue of how the stimulation parameters employed in the rodent research are related to those employed in the therapeutic intervention in humans. The pulse duration employed in the deep-brain stimulation of the human MFB is 60 s, which is even shorter than the 100 s duration typically employed in studies of intracranial self-stimulation in rats. Chronaxies of unmyelinated axons are typically longer than those of myelinated axons (West and Wolstencroft, 1983). Thus, the short pulse duration employed in deep-brain stimulation of the human MFB would render such stimulation even less likely to directly activate unmyelinated dopamine axons than the stimulation employed in the rodent studies.

The maximum firing frequency of human dopamine neurons has yet to be determined, as far as we know. That said, the pulse frequency employed in the deep-brain stimulation of the human MFB, 130 Hz, is well above the maximum firing frequency that dopaminergic neurons can sustain in the rodent (Tsai et al., 2009; Witten et al., 2011; Covey and Cheer, 2019).

### The Centrality of the Dopamine Neurons?

The papers detailing the antidepressant effect of MFB stimulation have consistently acknowledged the anatomical and neurochemical heterogeneity of the MFB (Coenen et al., 2011, 2012; Schlaepfer et al., 2013). However, after a tipping of the hat toward this incontestable neuroanatomical reality, the discussion in the early papers rapidly gravitates toward a dopamine-centered (“dopacentric”) view analogous to the series-circuit model in Figure 4. The authors recognize that the dopaminergic axons are less excitable to extracellular stimulation than the larger, myelinated MFB fibers interspersed among them. Thus, they have proposed that the directly stimulated elements subserving the rewarding effect are corticofugal afferents to VTA dopamine neurons (Schlaepfer et al., 2013, 2014; Coenen et al., 2021). These glutamatergic fibers excite their post-synaptic targets: the midbrain dopamine cells. The psychological and behavioral effects of the MFB stimulation are largely attributed to that excitation, as in the series circuit model. In the case of MFB self-stimulation in rats, the series-circuit model has been falsified by recent evidence (Trujillo-Pisanty et al., 2020) and fits poorly with an array of prior findings (Huston and Borbély, 1973; Pritzel et al., 1983; Johnson and Stellar, 1994; Cossette et al., 2016). This is what motivated the development of the convergence model (Figure 5 and Supplementary Figure 1) in which the activity of non-dopaminergic MFB fibers accessed the final common path for reward pursuit in parallel with the firing of midbrain dopaminergic neurons.

The failure of psychomotor stimulants to serve as an effective monotherapy for depression invites reconsideration of a series-circuit model of the antidepressant effect of MFB stimulation. An alternative, analogous to the convergence model of intracranial MFB self-stimulation, would include multiple, convergent pathways. On that view, non-dopaminergic MFB components may contribute to the therapeutic effect in parallel to, in synergy with, or even instead of, a dopaminergic component. To assess those possibilities, we must look in more detail at the neuroanatomical complexity of the region where MFB stimulation is effective in relieving treatment-resistant depression and at the methods that have been used to link that effect to particular fiber bundles.

### Which Neurons Are Activated Directly by Therapeutically Effective Stimulation of the Medial Forebrain Bundle, and Which Are Responsible for the Antidepressant Effect?

Evidence continues to accumulate that deep-brain stimulation of the MFB provides relief from depression that has resisted other forms of treatment (Schlaepfer et al., 2013; Fenoy et al., 2016, 2021; Coenen et al., 2018, 2019; Kilian et al., 2019). The effective stimulation site lies in a neuroanatomically complex region. Which of the local neural elements is directly activated by the electrical stimulation and gives rise to the therapeutic effect: local cell bodies, their afferents, fibers of passage, or some combination thereof?

The groups that are carrying out the neurosurgical work and following up on its consequences apply diffusion-weighted magnetic-resonance imaging tractography (“diffusion tractography”) to address this question (Thomas et al., 2014; Jbabdi et al., 2015; Maier-Hein et al., 2017). This non-invasive, inferential method is used extensively both in the surgical positioning of deep-brain stimulation electrodes and in interpreting the effects of the stimulation. It is based on the differential ease with which water molecules diffuse along and across fiber tracts. The volume elements (voxels) that constitute the spatial units of the structural MRI data from which the inferences about fiber trajectories are drawn are large compared to the diameters of individual axons, and multiple assumptions must be made in order to link the imaging data to its anatomical interpretation. As Haber et al. (2021) have noted: “multiple configurations of axon populations can give rise to similar diffusion profiles.”

The plausibility of findings obtained by means of diffusion tractography has been evaluated in non-human primates. In such studies, fiber tracts are visualized post-mortem by means of well-established neuroanatomical tract-tracing methods with high spatial resolution. The results are registered and compared to high-resolution, *ex vivo* diffusion tractography results obtained from the same subjects (Grisot et al., 2021). Such rigorous comparisons provide both good and not-so-good news: the two methods yield correspondence that is substantial but imperfect, particularly where projections from different sources cross, branch, abut, and/or bend (Grisot et al., 2021; Haber et al., 2021, 2022). One such location is the MFB in the vicinity of the VTA, the location of the MFB site where deep-brain stimulation can relieve treatment-resistant depression. Haber and colleagues note that:

“This complex midbrain area contains tightly packed intermixed myelinated bundles. As such, it likely modulates descending and ascending STN (sub-thalamic nucleus), ZI (zona incerta), and VTA/substantia nigra fibers entering and exiting the IC (internal capsule). The area also contains striato-brainstem, pallido-midbrain, cortico-brainstem, and hypothalamo-brainstem fibers” (Haber et al., 2021; acronym definitions added in parentheses).

The fibers coursing toward the brainstem are of particular interest given the evidence cited above for a reward-related pathway that parallels the midbrain dopamine projections (Trujillo-Pisanty et al., 2020), for reward-related fibers linking self-stimulation sites in the rat caudal to the midbrain dopamine neurons axonal to the VTA and lateral hypothalamus (Boye and Rompré, 1996), and for the view that the fundamental circuit subserving intracranial self-stimulation is located in the caudal brainstem (Huston, 1982).

Do the brain sections obtained to trace corticofugal fibers in the non-human primates contain additional information pertinent to identifying the neurons directly activated by electrical stimulation in humans that produces therapeutic effects? For example, how do the diameters and myelination of corticofugal fibers terminating in the VTA compare to those of corticofugal fibers that continue caudally as well as to those of brainstem-projecting fibers arising in the diencephalon and basal forebrain? Pertinent neuroanatomical methods for addressing such questions are addressed in a recent manuscript (Yendiki et al., 2021). Although a single hull is typically drawn around a therapeutically effective stimulation site to enclose the volume within which the stimulation triggers action potentials, a broad distribution of fiber diameters and myelination would require a Russian-doll-like depiction consisting of multiple concentric hulls, each corresponding to a different neural population defined on the basis of its excitability (Kringelbach et al., 2007). How does the appropriate Russian-doll-like depiction map onto the complex anatomy of the therapeutically effective MFB stimulation site?

Single-unit electrophysiology provides the most definitive means for determining whether particular neurons are directly excited by electrical stimulation. For example, the collision test (Bishop et al., 1962) establishes that an axon activated by a stimulation electrode at one brain site arises from a cell body in a second site. Can the pertinence of such studies in the non-human primate to identifying the directly stimulated neurons activated by MFB stimulation in humans be increased, perhaps by employing the same type of stimulation electrode and scaling the stimulation current to reflect the different dimensions of non-human-primate and human brains and axons? Could pertinent information be derived from post-mortem imaging of axons linking the stimulation and recording sites? Can such work tell us whether stimulation at the site homologous to the therapeutically effective locus in humans activates neurons that project to deep brainstem sites beyond the midbrain dopamine cell bodies?

In recognition of the differential excitability of dopaminergic axons and larger, myelinated fibers that also course through the therapeutically effective stimulation site, Coenen, Schlaëpfer and colleagues (referred to below as the “Freiburg group”) proposed that the antidepressant effect of MFB stimulation arises from the direct excitation of glutamatergic, corticofugal afferents to VTA dopamine neurons (Schlaepfer et al., 2013, 2014). In a recent paper (Coenen et al., 2021), their team aligned *in vivo* and *ex vivo* diffusion-tractography data, the latter acquired at higher resolution. The post-mortem specimen was stained to highlight nerve fibers and to visualize neurons expressing tyrosine-hydroxylase. The data were interpreted as support for the proposition that direct activation of corticofugal VTA afferents gives rise to the therapeutic effect. It is not clear whether this work puts to rest all the concerns voiced by the researchers who have compared diffusion tractography and neuroanatomical tracing methods in the non-human primate (Grisot et al., 2021; Haber et al., 2021, 2022). To their concerns, we add some questions of our own.

We wonder whether additional information about fiber-diameter spectra and axonal trajectories in the vicinity of the effective stimulation site could be extracted from the existing *ex vivo* human specimen or from additional such specimens examined by means of higher-resolution methods (Yendiki et al., 2021). There now appears to be agreement (Coenen et al., 2021; Haber et al., 2021, 2022) that the axons of the midbrain dopamine neurons in the human ascend in the classic MFB, as they do in the non-human primate, and that these axons do not join the internal capsule. Do we understand correctly that what the Freiburg group calls the superolateral MFB consists of contifugal fibers that occupy a quadrant of the anterior limb of the internal capsule and reach the VTA via the lateral hypothalamus? If so, to what degree are these fibers intermingled with those of the classic MFB en route from the lateral hypothalamus to the VTA (Coenen et al., 2021)? Is the VTA their sole terminal field, or are there branches or sub-components of the bundle that continue caudally?

Although dopaminergic activation is central to their account of the antidepressant effect of MFB stimulation, the Freiburg group has also considered another impact on cortical functioning, one due to antidromic propagation of the stimulation-induced firing of corticofugal fibers (Coenen et al., 2021). Presumably, the antidromic action potentials could invade cortical collaterals that drive local inhibitory interneurons. That proposal is of particular interest given a recent report linking maladaptive stress-induced glutamatergic responses in the medial prefrontal cortex in depressed patients to pessimistic expectations (Cooper et al., 2021). That said, the report from the Freiburg group emphasizes orbitofrontal efferents, rather than medial prefrontal ones. Electrophysiological data from non-human primates obtained using electrodes and stimulation sites homologous to the ones employed in the human clinical work would be of particular interest in this regard.

## Conclusion

In many of the papers describing their pioneering work on the use of MFB stimulation in humans to relieve treatment-resistant depression, the Freiburg group has tied their analysis to longstanding research on intracranial self-stimulation of the rodent MFB. Jaak Panksepp is a co-author of several of the early papers (Coenen et al., 2009b,^2011^, ^2012^), which adopt his qualitative perspective. When viewed through the lens of Panksepp’s SEEKING system, the midbrain dopamine neurons are *primi inter pares* among the constituents of the MFB that subserve self-stimulation. Outside the field of view of this lens is over 40 years of parallel quantitative work implicating non-dopaminergic components of the MFB in reward and appetitive motivation (e.g., Gallistel et al., 1981; Yeomans, 1990; Shizgal, 1997). The series-circuit hypothesis attempts to reconcile the dopacentric, qualitative view with the quantitative, psychophysical and electrophysiological work. However, the series-circuit hypothesis has foundered following the incorporation of optogenetic methods into the quantitative approach (Trujillo-Pisanty et al., 2020). The new convergence model arises from that work. In that model, the directly activated, non-dopaminergic fibers access the final common path for reward pursuit via circuitry that partially parallels the dopaminergic projections. On that view, the midbrain dopamine neurons remain vitally important, but they have company in the form of a parallel route to the behavioral final common path. We speculate on how emerging methods will lead to the identification of the parallel pathway, and we make a plea to keep interpretative filters open in evaluating potential contribution to the antidepressant effect of MFB stimulation by non-dopaminergic fibers coursing through or near the effective stimulation site. We also question why, if the deep-brain stimulation produces its therapeutic benefit by “tuning up” the ascending dopaminergic pathways (Döbrössy et al., 2021), does administration of psychomotor stimulants fall short of achieving the same ends?

In agreement with the Freiburg group and Panksepp, we hold that research on MFB self-stimulation in rodents will continue to have translational implications. We hope that future research into this seminal phenomenon, coupled with allied experimental work in non-human primates and humans, will yield a fuller understanding, both of the psychological and neural mechanisms underlying the antidepressant effect of deep-brain stimulation, and of the neural foundations of reward and motivation.

## Data Availability Statement

This manuscript refers to a publicly available dataset. This data can be found here: https://spectrum.library.concordia.ca/id/eprint/986807/.

## Ethics Statement

The animal study that generated the publicly available dataset was reviewed and approved by the Animal Research Ethics Committee, Concordia University.

## Author Contributions

VP and PS: conceptualization and writing. Both authors contributed to the article and approved the submitted version.

## Conflict of Interest

The authors declare that the research was conducted in the absence of any commercial or financial relationships that could be construed as a potential conflict of interest.

## Publisher’s Note

All claims expressed in this article are solely those of the authors and do not necessarily represent those of their affiliated organizations, or those of the publisher, the editors and the reviewers. Any product that may be evaluated in this article, or claim that may be made by its manufacturer, is not guaranteed or endorsed by the publisher.
