# Supplementary figures and images for: The Convergence Model of Brain Reward Circuitry: Implications for Relief of Treatment-Resistant Depression by Deep-Brain Stimulation of the Medial Forebrain Bundle
Source: Front Behav Neurosci. 2022 Apr 1;16:851067. doi: 10.3389/fnbeh.2022.851067 (PMC9011331; doi:10.3389/fnbeh.2022.851067)

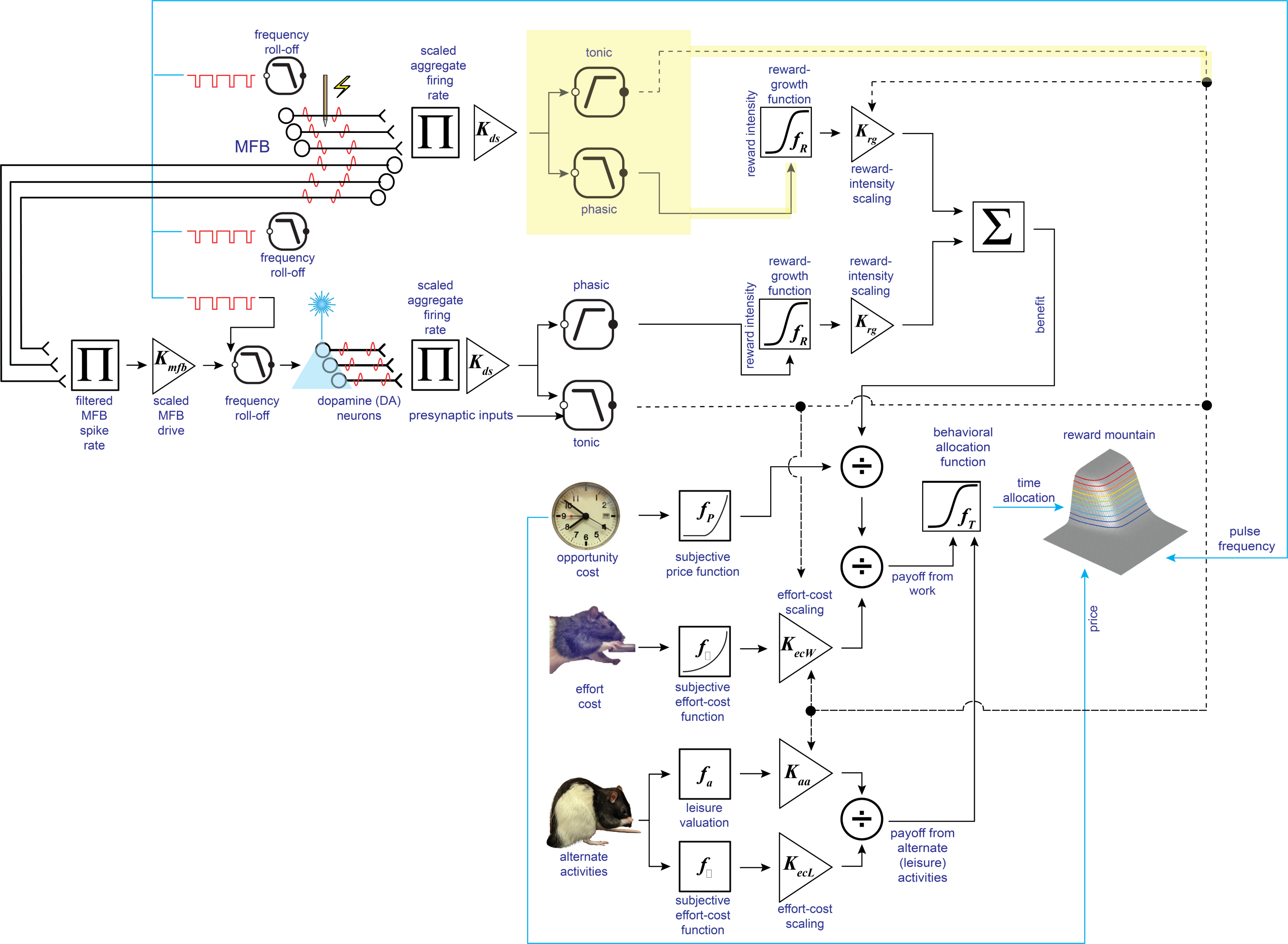

Supplement: Supplementary file 1 [file Image_1.pdf]
